# Evaluating the Clinical Effectiveness of Cardiac Rehabilitation among Patients of Very Low Socioeconomic Status Living in Colombia

**DOI:** 10.3390/jcdd11090255

**Published:** 2024-08-23

**Authors:** Gabriela L. M. Ghisi, Ana Paula Delgado Bomtempo, Nelson F. Gonzalez, Giovanna Patricia Reyes, Claudia V. Anchique

**Affiliations:** 1 KITE Research Institute, University Health Network, University of Toronto, Toronto, ON M4G 1R7, Canada; 2 Graduate Program in Physical Education, Faculty of Physical Education and Sports, Federal University of Juiz de Fora, Juiz de Fora 36036-900, Brazil; ana.dbomtempo@gmail.com; 3 Mediagnostica Tecmedi S.A.S., Duitama 150007, Colombia

**Keywords:** cardiac rehabilitation, low socioeconomic status, cardiovascular disease, low- and middle-income country, health disparities, exercise capacity, quality of life

## Abstract

Cardiovascular disease (CVD) poses a significant health burden, particularly among individuals of low socioeconomic status (SES) in low- and middle-income countries (LMICs). This study evaluates the clinical effectiveness of cardiac rehabilitation (CR) in addressing CVD outcomes among very low-SES patients in Colombia. Data from participants enrolled in a CR program in Colombia between 2022 and 2023 were analyzed retrospectively. Measures included heart-healthy behaviors, physical/psychological outcomes, and quality of life assessed at 18, 36, and 60 sessions. Significant improvements were observed in exercise capacity, psychological well-being, and quality of life metrics throughout the CR program. However, barriers to CR attendance and the critical need for expanded program availability remain evident, particularly in LMIC settings like Colombia. In conclusion, structured CR programs demonstrate substantial benefits for very low-SES individuals in a LMIC country, highlighting the urgent need for increased program accessibility and equitable healthcare provision to optimize cardiovascular health outcomes.

## 1. Introduction

Cardiovascular disease (CVD) is the leading cause of morbidity and mortality worldwide [1], and a major contributor of premature disability and economic impact on a global scale [2,3]. The burden of CVD is particularly high in those living with low socioeconomic status (SES) in low- and middle-income countries (LMICs) [4,5]. SES significantly impacts cardiovascular health, with lower SES individuals experiencing higher event rates and worse outcomes [6,7]. In addition, LMICs are responsible for 80% of global cardiovascular deaths [1]. Of particular concern is that premature deaths account for approximately half of all fatalities in developing nations. Despite this burden, LMICs contribute only 2.8% of CVD research output while accounting for 59.5% and 57.1% of global CVD disability-adjusted life years lost and death rates, respectively [8]. The combination of low SES and the high prevalence of CVD in LMICs underscores a critical global health disparity requiring urgent attention and targeted interventions.

Cardiac rehabilitation (CR) plays a pivotal role in mitigating this global burden. It serves as a comprehensive structured program designed to optimize cardiovascular health and quality of life for individuals who have experienced a cardiac event or undergone cardiac surgery [9]. Typically, CR programs encompass supervised exercise training, education on heart-healthy lifestyles, dietary guidance, psychosocial support, and risk factor management [9,10]. These interventions have been well established to improve exercise capacity, reduce hospitalizations, enhance psychological well-being, and lower mortality rates among participants [11,12], including those in LMICs [13].

Despite the well-documented benefits of CR, it remains underutilized globally [14]. Low SES is often linked to lower rates of referral and participation in CR programs [15]. Moreover, there is a notable lack of research exploring how CR specifically impacts individuals with low SES in LMICs. This paucity of research hinders efforts to address disparities and optimize cardiovascular health outcomes in vulnerable populations. Research also indicates that CR programs are available in only 22.1% of LMICs [16], with referral rates below 40% and high dropout rates [17]. In Colombia, where the burden of CVD is substantial and access to CR is limited, only 50 centers offer these services [18], and those that do often face challenges with patient engagement and retention [19]. Therefore, this study aimed to assess the clinical effectiveness of CR among patients with very low SES living in Colombia, an LMIC with a significant CVD burden, to explore effective interventions for underserved populations in the region [20,21].

## 2. Materials and Methods

This retrospective study analyzed outcomes from individuals with very low SES enrolled in a 6-month CR program at Mediagnostica Tecmedi (Colombia) between January 2022 and December 2023. Data were collected at 18, 36, and 60 sessions. On average, 18 sessions take 6 to 10 weeks, 36 sessions take 14 to 24 weeks, and 60 sessions take 30 to 50 weeks to complete. The study received approval from the local hospital Research Ethics Board (01-2024).

### 2.1. Setting

This study was conducted at the CR program at Mediagnostica Tecmedi in Colombia. The program is designed as a comprehensive outpatient chronic disease management model, following clinical practice guidelines [9,22]. The program is led by an interdisciplinary team comprising a cardiologist, a sports medicine specialist, physiotherapists, nursing staff, and an administrative group. It covers the following CR core components: exercise training and physical activity counseling, weight management and nutritional guidance, cardiovascular risk factor management, medication management and adherence, stress management, depressive symptom management, social support networking, and patient education. Cardiovascular risk factor management includes promoting weight control to achieve a healthy BMI, managing lipid profiles, frequent blood pressure monitoring, and encouraging smoking cessation with support for quitting. Lifestyle changes and treatment strategies are recommended for those with elevated waist circumference or associated risk factors such as hypertension or diabetes.

Sessions are conducted in a group setting with class sizes typically ranging from 15 to 20 participants. Each exercise session consists of 30 min of structured aerobic exercise, 15 min of resistance training, and 15 min of flexibility exercises. These sessions are tailored based on individualized prescriptions from a rehabilitation physician, taking into account each participant’s physical abilities and/or limitations. The educational component includes group lectures held every two weeks, covering topics such as medications, symptoms, healthy lifestyles, exercise, nutrition, and cardiovascular risk factors. Additionally, group workshops are conducted bi-weekly, focusing on nutrition, stress management, coordination exercises, and flexibility. Participants receive individual education and feedback on specific topics during these sessions. To further promote healthy living, leisure activities with an emphasis on healthy lifestyles are organized every three months.

### 2.2. Participants

Participants enrolled from 2022 to 2023 who were classified as belonging to very low estratos (strata) according to Colombia’s Class System [21] were eligible for inclusion. Colombia’s class system categorizes urban households into socioeconomic levels, influencing access to services and resources across the country. The three lowest SESs—included in this study—were the following:

- Stratum I (Bajo Bajo): The lowest socioeconomic level, consisting of households with the fewest economic resources. Residents in stratum I often face significant challenges in accessing basic services such as water, sanitation, and electricity due to limited affordability.
- Stratum II (Bajo): The second-lowest stratum includes households with somewhat improved but still modest economic means compared to the national average. Residents in stratum II typically have access to basic services but may encounter difficulties in affording higher-quality amenities and services.
- Stratum III (Medio Bajo): This stratum comprises households with moderate economic resources that are below the national average. Residents in stratum III generally have better access to services compared to stratum I and II, including more reliable utilities and infrastructure, although they may still experience socioeconomic challenges.

### 2.3. Clinical Characteristics

Clinical characteristics, including CR referral indication, cardiac risk factors, and comorbidities, were extracted from medical records, and defined according to guidelines [9,22]:

- Dyslipidemia: A condition characterized by abnormal lipid levels in the blood. Optimal levels for lipids are LDL cholesterol less than 100 mg/dL, HDL cholesterol more than 40 mg/dL for men and more than 50 mg/dL for women, triglycerides less than 150 mg/dL, and total cholesterol less than 200 mg/dL.
- Abdominal obesity: Excess fat stored around the abdomen, often measured by waist circumference (≥94 cm for men and ≥80 cm for women) or waist-to-hip ratio (≥0.9 for men and ≥0.85 for women).
- Sedentary: A pattern of behavior involving prolonged periods of sitting, reclining, or lying down, characterized by an energy expenditure of ≤1.5 metabolic equivalents (METs) while awake.
- Hypertension: A condition defined by consistently elevated blood pressure readings of 130/80 mm Hg or higher.
- Stress: A state where an individual’s organs or systems face demands exceeding normal performance, requiring adaptations that may lead to physical and emotional responses. These adaptations, which can be acute or chronic, include anxiety, emotional exhaustion, and social isolation, potentially contributing to conditions like depression.
- Overweight: A condition where an individual has more body fat than is considered healthy, typically defined by a BMI ranging from 25 to 29.9 kg/m^2^.
- Diabetes: A condition diagnosed when the average blood sugar level, measured using the hemoglobin A1c test, is 6.5% or higher on two separate tests.
- Obesity: A condition characterized by excessive body fat accumulation, typically defined by a body mass index (BMI) of 30 kg/m^2^ or greater.
- Prediabetes: A condition where the average blood sugar level is between 5.7% and 6.4% on the hemoglobin A1c test, indicating an increased risk of developing diabetes.
- Alcohol abuse: For men, consuming five or more drinks on any day or fifteen or more drinks per week. For women, consuming four or more drinks on any day or eight or more drinks per week.
- Past smoker: Someone who has smoked at least 100 cigarettes in his or her lifetime but who had quit smoking at the time of interview.
- Current smoker: Someone who has smoked 100 cigarettes in his or her lifetime and who currently smokes cigarettes.
- Passive smoker: An individual exposed to tobacco smoke from others.

### 2.4. Sociodemographic Characteristics

Sociodemographic information such as occupation, highest educational attainment, and marital status was self-reported by patients. Additionally, patients indicated family support (yes/no) and rated emotional support using a Likert-type scale ranging from 1 to 5, where higher scores indicated greater perceived support. Heart-healthy behaviors, physical/psychological outcomes, and quality of life were assessed at baseline and subsequently at 18 (T1), 36 (T2), and 60 (T3) sessions of CR.

### 2.5. Measures

Heart-healthy behaviors were predominantly self-reported, supplemented by data from pedometers. Specifically, participants’ daily steps, the number of active weekdays per week (i.e., days of the week that the individual engaged in physical activity or exercise), and servings of fruits and vegetables consumed daily were assessed as indicators of their adherence to heart-healthy practices. Pedometers provided objective measures of physical activity [23], complementing self-reported data to offer a comprehensive view of participants’ behavior patterns related to cardiovascular health. Participants were instructed to wear the pedometer on their hip continuously for 7 days, ideally consecutively leading up to each assessment point.

Physical outcomes were assessed via the 6-Minute Walk Test (6MWT) distance [24], METS (Metabolic Equivalents of Task; calculated via 6MWT), and the Downton Fall Risk scale [25]. The 6MWT distance provided an objective measure of aerobic endurance and functional capacity, with participants walking as far as possible in six minutes [24]. METS quantified the intensity of physical activities relative to resting metabolic rate, offering insight into participants’ exercise tolerance [26]. Additionally, the Downton Fall Risk scale evaluated fall risk, assigning points based on specific criteria to assess participants’ likelihood of falling, thereby contributing to a comprehensive assessment of physical health and mobility outcomes. Those who scored more than 3 points were classified as “High Risk” on the Fall Risk Scale [25].

Psychological outcomes were assessed using the Patient Health Questionnaire-9 (PHQ-9) [27]. This instrument is used to screen and measure the severity of depression. It consists of nine questions scored from 0 to 3, representing an increasing severity of symptoms, with total scores ranging from 0 to 27. Higher scores indicate more severe depressive symptoms.

Finally, quality of life was assessed using two measures: Cadril’s ladder scale [28] and the Short Form 36 (SF-36) questionnaire [29]. The ladder scale, a visual analog scale, asks participants to rate their overall quality of life on a scale from 0 to 10, with higher scores indicating better perceived quality of life [28]. The SF-36 questionnaire is a comprehensive tool that evaluates multiple dimensions of health-related quality of life, including physical functioning, role limitations due to physical health problems, bodily pain, general health perceptions, vitality, social functioning, role limitations due to emotional problems, and mental health [29]. Scores for each dimension are aggregated into physical and mental health summary scores, providing a detailed assessment of participants’ perceived health status and well-being across various domains. Scores for each component range from 0 to 100, with higher scores indicating better health-related quality of life.

### 2.6. Data Analysis

Data analysis was performed using the Statistical Package for Social Sciences v.28 (SPSS Inc., Chicago, IL, USA). The normality of continuous variables was assessed using Shapiro–Wilk tests. Initially, participant characteristics were analyzed descriptively, using medians with interquartile ranges due to non-normally distributed data, as well as frequencies and percentages. To evaluate changes over time across the baseline, 18, 36, and 60 sessions, the Wilcoxon signed-rank test was applied due to the non-normal distribution of the data. Bonferroni correction was implemented to adjust for multiple comparisons, setting the threshold for statistical significance at a *p*-value of 0.005.

## 3. Results

### 3.1. Characteristics of Participants

Of the 605 patients who participated in the CR program between 2022 and 2023, 549 (91.0%) were from very low strata (strata I to III) and were included in this study. The characteristics of these participants are detailed in Table 1. As shown, the average age was 65 years old, with the cohort nearly evenly split by age, comprising 49.7% under 65 and 50.2% aged 65 or older. The majority were male (58.3%). In terms of educational status, 37.7% had completed elementary school, while only 17.5% had a university degree. Most participants were married or in an equivalent relationship (65.6%), and 43% were employed. Clinically, the primary CR indications were heart failure (93.8%; with 90.0% of them with heart failure with preserved ejection fraction) and coronary artery disease (64.3%). Common comorbidities included dyslipidemia (77.6%), abdominal obesity (76.5%), and hypertension (66.5%). On average, participants attended 36 sessions.

### 3.2. Effectiveness of CR

Table 2 displays the scores of outcomes of interest at baseline and subsequently at 18, 36, and 60 sessions of CR. Heart-healthy behaviors, including daily steps, the number of active weekdays per week, and servings of fruits and vegetables consumed daily, showed significant improvements from baseline to the subsequent time points T1, T2, and T3. Participants’ daily steps increased significantly from a median of 5555 at baseline to 7420 at T1, further rising to 8182 at T2 and reaching 9181 at T3 (*p* < 0.001). The number of active weekdays per week also saw notable gains, starting at a median of 0 days at baseline, jumping to 5 days at T1, maintaining stability at T2, and slightly increasing to 5.5 days at T3 (*p* < 0.001). Similarly, the daily consumption of fruits and vegetables improved, with servings increasing from a median of three at baseline to four at T1, remaining at four at T2, and climbing to five at T3 (*p* < 0.001). These enhancements in heart-healthy behaviors were statistically significant across all assessment points, indicating substantial positive changes in lifestyle habits over the course of the CR program.

In regard to physical outcomes, the 6MWT distance showed significant improvements, with participants increasing their median distance from 396 m at baseline to 469 m at T1, 510 m at T2, and 512 m at T3 (*p* < 0.001). METs also demonstrated significant gains, starting at a median of 4.0 at baseline, rising to 4.5 at T1 and 4.8 at T2, and remaining at 4.8 at T3 (*p* < 0.001). The Fall Risk Scale, however, did not show significant changes, with participants maintaining a median score of 2 from baseline through all time points (*p* = 0.15). These findings indicate substantial improvements in exercise capacity and physical fitness, as evidenced by the 6MWT distance and METs.

In regard to psychological outcomes, participants showed a significant reduction in their PHQ-9 scores, indicating an improvement in psychological well-being. The median score decreased from 3 at baseline to 1 at T1 (*p* < 0.001), with no further reduction at either T2 or T3. Across assessments from baseline to T3, there was a notable decrease in moderate (6.6% to 0.9%), moderately severe (3.1% to 1.9%), and severe depression (1.6% to 0.9%) severity, as classified by the PHQ-9 [23].

Finally, the two quality of life measures also improved significantly throughout the study period. For the ladder scale, participants reported a median score increase from 7 at baseline to 8 at T1, remaining at 8 at both T2 and T3 (*p* < 0.001). Similarly, the SF-36 questionnaire demonstrated significant enhancements in both physical and mental components of quality of life. The physical component score rose from a baseline median of 56 to 82 at T1, continuing to improve to 89 at T2 and 94.4 at T3 (*p* < 0.001). Likewise, the mental component score increased from 70 at baseline to 88 at T1, further improving to 93 at T2 and reaching 100 at T3 (*p* < 0.001).

## 4. Discussion

CR programs are vital in addressing the global burden of CVD, particularly among individuals with low SES living in LMICs [13,30]. This study aimed to evaluate the clinical effectiveness of CR among patients from very low SES backgrounds in Colombia, a country grappling with significant CVD prevalence and health disparities. Our findings underscore the transformative impact of structured CR interventions on heart-healthy behaviors, physical and psychological outcomes, and quality of life among underserved populations. Across a comprehensive assessment period, participants demonstrated remarkable improvements significantly and rapidly, from T1, and then increased or sustained in the other time points (T2 and T3), highlighting CR’s potential to mitigate disparities and enhance cardiovascular health in vulnerable communities.

Despite the evident benefits of CR programs, individuals from low SES backgrounds in LMICs often face significant barriers to attendance and participation. Financial constraints, limited access to transportation, and competing responsibilities such as work and caregiving duties frequently deter participation [31,32,33]. Moreover, cultural beliefs and perceptions about illness and rehabilitation may influence willingness to engage in structured health interventions [33]. Addressing logistical, financial, and cultural obstacles and ensuring access to comprehensive CR programs can facilitate greater engagement and optimize health outcomes among vulnerable populations.

Colombia, like many LMICs, grapples with a high burden of CVD exacerbated by socioeconomic disparities and limited access to healthcare resources [34,35]. Despite these challenges, the presence of structured CR programs represents an important step towards addressing these health inequities. Currently, the country hosts approximately 50 CR programs, resulting in a CR density of one spot for every four patients in need [36]. However, there remains a substantial gap, as over 55,000 additional spots are required to adequately support all patients in need of CR services [36]. This study underscores the critical importance of CR programs by demonstrating their positive impact on cardiovascular health outcomes among individuals from very low socioeconomic backgrounds. While the results highlight the effectiveness of structured CR interventions, the need for expanded program availability is evident [37,38]. Addressing this gap is essential to ensuring equitable access to CR and improving health outcomes for all Colombians affected by CVD, thereby contributing both locally and globally to reducing health disparities and enhancing quality of life in LMICs.

In our study, we did not find significant results regarding fall risk among participants. However, it is crucial to emphasize the importance of routinely measuring fall risk during CR programs [39]. The literature consistently underscores that individuals undergoing CR, especially those with cardiovascular conditions and older adults, are at an increased risk of falls due to factors such as reduced muscle strength, balance deficits, and medication side effects [40,41]. Effective strategies to prevent falls in this population include tailored exercise programs that improve strength and balance, patient education on fall prevention strategies, and environmental modifications [41]. Integrating fall risk assessment and prevention measures into CR protocols not only enhances patient safety but also contributes to improved overall health outcomes and quality of life for participants. Future research and practice should continue to prioritize comprehensive approaches that address fall prevention as an integral component of CR.

The attendance rate in our study was remarkably high, with patients attending between 50% and 75% of the possible sessions. Notably, over 40% of these participants were employed and came from very poor socioeconomic backgrounds, yet they still managed to participate in the CR program. Typically, patients from lower SES backgrounds attend fewer CR sessions compared to their higher-SES counterparts and may not achieve the same level of benefit from participation [42]. The specific challenges faced by this cohort, such as financial constraints and limited access to transportation, often necessitate tailored interventions to ensure consistent participation. Despite this positive trend, our study’s design limits our ability to explore the barriers and facilitators influencing CR attendance in depth. Other studies have identified several key factors affecting attendance, including patient motivation, the perceived value of the program, and logistical issues [43]. Their findings suggest that while socioeconomic factors play a role, individual and systemic barriers, such as transportation and scheduling conflicts, also significantly impact attendance rates. Our study’s results highlight the commitment of participants despite their challenging circumstances, underscoring the potential benefits and importance of CR programs. This commitment is consistent with other studies that patients who perceive significant personal benefits from CR programs are more likely to overcome barriers and maintain high attendance rates [44,45]. This observation suggests that enhancing patient engagement and demonstrating the value of CR programs could further improve adherence.

Our study found that participants from very low socioeconomic backgrounds reported high quality of life and relatively low levels of depressive symptoms. Typically, cardiac patients with low SES are 3 to 5 times more likely to experience poor psychological well-being, worse mental health status, and lower quality of life compared to those with higher SES [42,46]. The high quality of life scores in our study could be attributed to the comprehensive support provided by the CR program [15], including physical exercise, dietary counseling, and psychological support. Additionally, the positive impact of social support and the sense of accomplishment from participating in a structured CR program likely contributed to the observed improvements in quality of life and lower PHQ-9 scores. There is a need for further research to explore the specific barriers and facilitators of CR participation among very low socioeconomic populations and to assess the broader impact of CR programs on various patient groups. This will be essential for refining CR interventions to ensure they are accessible and effective for all patients, especially those from disadvantaged backgrounds.

The findings of this study carry important implications for policy and practice in cardiovascular rehabilitation, particularly in LMICs like Colombia. Demonstrating the efficacy of structured CR programs in improving heart-healthy behaviors, physical and psychological outcomes, and quality of life underscores the urgent need for expanded access to such interventions. Policymakers should prioritize the development and scaling of CR programs tailored to meet the needs of underserved populations, including those from low socioeconomic backgrounds. Future research should focus on evaluating the scalability and sustainability of CR interventions across diverse settings, as well as exploring innovative strategies to enhance program accessibility and participation rates. By addressing these challenges, healthcare systems can effectively mitigate health disparities and improve cardiovascular health outcomes on a broader scale.

Several limitations should be considered when interpreting the findings of this study. Firstly, the retrospective design and reliance on self-reported data for heart-healthy behaviors may introduce recall bias and affect data accuracy. Additionally, the study’s focus on participants from a single CR program in Colombia limits the generalizability of findings to broader populations and settings within the country. Moreover, while efforts were made to include participants from very low socioeconomic backgrounds, variations in participant characteristics and program implementation across different CR settings may influence the reproducibility of results in other contexts.

## 5. Conclusions

In conclusion, this study provides compelling evidence of the substantial benefits that structured CR programs can offer to individuals from very low socioeconomic backgrounds in Colombia. The positive impacts observed in heart-healthy behaviors, physical and psychological outcomes, and quality of life underscore the pivotal role of CR in mitigating the burden of cardiovascular disease and improving overall well-being. While this study did not specifically assess barriers to CR attendance and participation, its findings suggest that expanding program availability and ensuring equitable access across Colombia and similar LMICs are crucial steps towards maximizing the potential benefits of CR interventions. Addressing these challenges requires concerted efforts from policymakers, healthcare providers, and communities to ensure that all individuals, regardless of SES, can access and benefit from CR programs. By prioritizing investment in comprehensive CR initiatives and implementing inclusive strategies, healthcare systems can advance towards achieving more equitable health outcomes and enhancing the cardiovascular health of populations worldwide.

## Figures and Tables

**Table 1:** Sociodemographic and clinical characteristics of male and female participants at baseline (N = 546).

Characteristic	Total (N = 546)
*Sociodemographic characteristics*	
Age	65 [55.2–72.0]
<65 years old	273 (49.7%)
≥65 years old	275 (50.2%)
Sex	
Male	320 (58.3%)
Female	229 (41.7%)
Highest education	
Elementary school	207 (37.7%)
Secondary school	153 (27.9%)
College	68 (12.4%)
University	96 (17.5%)
Graduate degree	10 (1.3%)
Marital status	
Married or equivalent	360 (65.6%)
Not married (single, divorced, widowed)	189 (34.4%)
Occupation	
Employed	236 (43.0%)
Household	168 (30.6%)
Retired	110 (20.0%)
Student	28 (5.1%)
Unemployed	4 (0.7%)
Socioeconomic class ^1^	
Class I	92 (16.8%)
Class II	301 (54.8%)
Class III	156 (28.4%)
Family support, yes	516 (94.0%)
Emotional support ^2^	5 [4–5]
*Clinical characteristics*	
CR indicational	
Heart failure	515 (93.8%)
Coronary artery disease	353 (64.3%)
PCI	219 (39.9%)
Myocardial infarction	201 (36.6%)
Metabolic syndrome	68 (12.4%)
Syncope	57 (10.4%)
CABG	47 (8.6%)
Valve disease	48 (8.7%)
Arrhythmias or ablation	43 (7.8%)
Unstable angina	41 (7.5%)
CSFP	30 (5.5%)
Implantable electrical stimulation devices	30 (5.5%)
Stable angina	21 (3.8%)
Coronary microvascular disease	17 (3.1%)
PAD	10 (1.8%)
Congenital heart disease	9 (1.6%)
MINOCA	9 (1.6%)
Post-COVID syndrome	9 (1.6%)
INOCA	6 (1.1%)
ANOCA	5 (0.9%)
Cerebrovascular disease	4 (0.7%)
Pulmonary disease	2 (0.4%)
Comorbidities	
Dyslipidemia	426 (77.6%)
Abdominal obesity	420 (76.5%)
Sedentary	417 (76.0%)
Hypertension	365 (66.5%)
Stress	338 (61.6%)
Overweight	242 (44.1%)
Diabetes	135 (24.6%)
Obesity	132 (24.0%)
Prediabetes	30 (5.5%)
Alcohol abuse	16 (2.9%)
Smoking history	
Past smoker	158 (28.8%)
Current smoker	24 (4.4%)
Passive smoker	14 (2.6%)
Attended sessions	36 [15–76]

Abbreviations: ANOCA, angina with no obstructive coronary artery disease; CABG, coronary artery bypass graft; CR, cardiac rehabilitation; CSFP, coronary slow flow phenomenon; INOCA, ischemic and non-obstructive coronary arteries; MINOCA, myocardial infarction with non-obstructive coronary arteries; PAD, peripheral arterial disease; PCI, percutaneous coronary intervention. Note: n (valid %), median [interquartile interval] shown. ^1^ The social class system in Colombia is classified into six strata. Only strata I, II, and III are reported as per the inclusion criteria of this study. ^2^ rated on a 5-point Likert-type scale, such that higher scores denote greater endorsement of the given construct.

**Table 2:** Impact of comprehensive CR intervention on outcomes of interest by assessment point (N = 546).

Outcome	Maximum Score	Baseline	T1	Change ^1^	T2	Change ^1^	T3	Change ^1^	*p* ^2^
Active days per week	7	0 [0–4]	5 [3–6]	+5 ***	5 [4–6]	0	5.5 [5–6]	+0.5	<0.001
Depression (PHQ-9)	27	3 [1–5]	1 [0–3]	−2.00 ***	1 [0–2]	−1 ***	1 [0–2]	0	<0.001
Distance 6MWT	-	396 [329–458]	469 [401–531]	+100 ***	510 [431–558]	+41 ***	512 [445–577]	+2 ***	<0.001
Fall risk	15	2 [1–3]	2 [1–3]	0	2 [1–3]	0	2 [2–4]	0	0.15
METs	-	4.0 [3.6–4.4]	4.5 [4.0–4.9]	+0.50 ***	4.8 [4.2–5.1]	+0.3 ***	4.8 [4.3–5.2]	0	<0.001
Servings of fruits and vegetables per day	-	3 [2–4]	4 [3–5]	+1 ***	4 [3–5]	0	5 [4–5]	+1	<0.001
Steps per day	-	5555 [3479–8139]	7420 [5176–9971]	+1865 ***	8182 [5778–10,515]	+762 ***	9181 [6253–11,877]	+999 ***	<0.001
Quality of life—ladder	10	7 [6–8]	8 [8–9]	+1 ***	8 [8–9]	0	9 [8–9]	+1	<0.001
Quality of life—SF-36	100	63 [45–76]	86.5 [75–93]	+23.5 ***	92.5 [84.7–97]	+6 ***	96 [87.8–99]	+3.5 ***	<0.001
Physical component	100	56 [41–70]	82 [69–91]	+26 ***	89 [80–95]	+7 ***	94.4 [84.8–98.4]	+5.4 ***	<0.001
Mental component	100	70 [51–84]	88 [78–94]	+18 ***	93 [86–96]	+5 ***	100 [92–100]	+7 ***	<0.001

Abbreviations: 6MWT, six-minute walking test; PHQ-9, patient health questionnaire; SF-36, the 36-item short form survey. T1: 18-session assessment; T2: 36-session assessment; T3: 60-session assessment. ^1^ Wilcoxon signed-rank test with Bonferroni correction *** *p* < 0.005. ^2^ Wilcoxon signed-rank test with Bonferroni correction between baseline and 60 sessions.

## Data Availability

Data are available upon request. Please contact the corresponding author.
